# The Biological Function Delineated Across Pan-Cancer Levels Through lncRNA-Based Prognostic Risk Assessment Factors for Pancreatic Cancer

**DOI:** 10.3389/fcell.2021.694652

**Published:** 2021-06-14

**Authors:** Xudong Tang, Mengyan Zhang, Liang Sun, Fengyan Xu, Xin Peng, Yan Zhang, Ying Deng, Shuliang Wu

**Affiliations:** ^1^Department of Human Anatomy, Harbin Medical University, Harbin, China; ^2^Computational Biology Research Center, School of Life Sciences and Technology, Harbin Institute of Technology, Harbin, China; ^3^Department of Emergency Medicine, The Second Affiliated Hospital of Harbin Medical University, Harbin, China

**Keywords:** lncRNA, prognostic model, pancreatic cancer, pan-cancer, survival analysis

## Abstract

Long non-coding RNAs (lncRNAs) play key roles in tumors and function not only as important molecular markers for cancer prognosis, but also as molecular characteristics at the pan-cancer level. Because of the poor prognosis of pancreatic cancer, accurate assessment of prognosis is a key issue in the development of treatment plans for pancreatic cancer. Here we analyzed pancreatic cancer data from The Cancer Genome Atlas and The Genotype Tissue Expression database using Cox regression and lasso regression in analyses using a combination of the two databases as well as only The Cancer Genome Atlas database (Cancer Genome Atlas Research Network et al., 2013). A prognostic risk score model with significant correlation with pancreatic cancer survival was constructed, and two lncRNAs were investigated. Additional analysis of 33 cancers using the two lncRNAs showed that lncRNA TsPOAP1-AS1 was a prognostic marker of seven cancers, among which pancreatic cancer was the most significant, and lncRNA mi600hg was a prognostic marker of ovarian cancer and pancreatic cancer. LncRNA TsPOAP1-AS1 is associated with clinical stage and tumor mutation burden of some cancers as well as a strong degree of immune infiltration in many cancers, while a strong correlation between lncRNA mi600hg and microsatellite instability was observed in several cancers. The results of this study help further our understanding of the different functions of lncRNAs in cancer and may aid in the clinical application of lncRNAs as prognostic factors for cancer.

## Introduction

Early research on pancreatic cancer has made some progress, and several pancreatic cancer-related genes have been discovered (He et al., 2014; Wolpin et al., 2014). However, comprehensive understanding of the mechanisms underlying pancreatic cancer remains limited. Recent research has suggested that long non-coding RNAs (lncRNAs) may provide new insights and help further pancreatic cancer research.

LncRNAs are transcripts >200 nucleotides in length that are present in various locations in the genome, including between genes and in promoters, enhancers and introns. LncRNAs have been the subject of investigation in recent years, and studies have demonstrated that lncRNAs are involved in multiple biological activities. Although the lncRNAs studied so far account for only a small portion of the total number of lncRNAs in the genome, these lncRNAs have been linked to various diseases, especially tumors (Kung et al., 2013; Ren, 2020). Multiple studies have established that lncRNAs participate in tumorigenesis and development. Over recent years, research has shown that lncRNAs play important roles in the mechanism of the occurrence, evolution, invasion and metastasis of pancreatic cancer. LncRNAs have also shown great potential as diagnostic markers, drug targets and prognostic factors for survival analysis (Natoli and Andrau, 2012; Yu et al., 2012). Several studies have described the impact of some lncRNAs on the prognosis of patients with pancreatic cancer (Huang et al., 2016), and several prognostic risk models have been established. Zhou et al. established a prognostic model for pancreatic cancer containing five lncRNAs (RP11-159F24.5, RP11-744N12.2, RP11-388M20.1, RP11-356C4.5, and CTC-459F4.9) through analysis of The Cancer Genome Atlas (TCGA) data; this model provides the possibility for survival prediction of pancreatic cancer patients and selection of biological treatment targets (Zhou et al., 2019). Zhang et al. (2019) used TCGA data to construct a prognostic risk prediction model for pancreatic cancer patients containing 12 lncRNAs (CTC-429P9.3, CTD-2186M15.3, RP5-890O3.9, AP000254.8, RP5-1085F17. 3, LINC01089, lncRNAs: LINC00941, ABHD11-AS1, CASC8, CYTOR, MIR4435-2HG, and UCA1). This risk score model can be used as an independent predictor of the prognosis of pancreatic cancer patients (Zhang et al., 2019). Wei et al. (2019) also analyzed the sequencing and clinical data of pancreatic cancer patient samples in TCGA database and identified nine immune-related lncRNAs (AL138966.2, AL133520.1, AC142472.1, AC127024.5, AC116913.1, AC083880.1, AC124016.1, AC008443.5, and AC092171.5). The authors constructed a pancreatic cancer prognostic risk scoring model that can be used as a potential target for pancreatic cancer immunotherapy (Wei et al., 2019). However, these studies have several shortcomings, including limitations regarding data samples, algorithms and efficacy evaluations. Moreover, the studies did not apply their models to pan-cancer research or explore the function of the lncRNAs.

In this study, we aimed to construct a reliable prognostic lncRNA model for pancreatic cancer and a pan-cancer analysis. We analyzed the lncRNA expression profile of tumor tissues from 178 pancreatic cancer patients in TCGA data. Gene expression data from normal pancreas samples in the GTEx database were used to correct for the imbalance in the number of non-tumor and cancer samples. A pan-cancer analysis of important lncRNAs was carried out to explore the possibility of lncRNAs as prognostic molecular markers in multiple tumors.

## Materials and Methods

### Data Sources and Data Preprocessing

We obtained gene expression profiles and related clinical documents of 177 pancreatic cancer tumor tissues and 4 adjacent tissues from TCGA database^1^ and downloaded and processed gene expression profiles of 180 non-cancerous human pancreatic tissues from the GTEx official website^2^. The TCGA and GTEx datasets were divided into a training set and test set at a 5:5 ratio. We also downloaded gene expression data of 33 cancers from the UCSC Xena official website^3^ and used the GRCh38 version of the genome annotation file^4^ from the Ensembl database to annotate lncRNAs. We obtained the immune gene set from the ImmPort database^5^ for subsequent analysis.

### Prognostic Risk Scoring Model Construction

The expression data of candidate lncRNAs obtained after single-gene Cox proportional hazard analysis and optimization by lasso regression analysis. Then, multi-factor Cox regression analysis were used to construct a prognostic risk scoring model for lncRNA. The final formula is:

Risk⁢score=β⁢gene⁢(1)×exprgene⁢(1)+β⁢gene⁢(2)×exprgene⁢(2)+…+β⁢gene⁢(n)×exprgene⁢(n).Risk⁢score=β⁢gene⁢(1)×exprgene⁢(1)+β⁢gene⁢(2)×exprgene⁢(2)+…+β⁢gene⁢(n)×exprgene⁢(n).

### Co-expression Analysis of Key lncRNAs

The most significant lncRNAs were screened out from the established Cox prognostic risk scoring model, and Pearson’s correlation analysis was performed in the normalized protein coding gene (PCG) expression dataset of pancreatic cancer patients and key lncRNAs. The screening threshold was defined as the absolute value of the correlation coefficient |Cor| > 0.4, *P* < 0.001. Based on these genes, gene function enrichment analysis was performed to predict the possible biological and cell signal transduction pathways of the candidate lncRNAs.

### GO Function Enrichment and KEGG Signal Pathway Analysis

We used the clusterProfiler package (v.3.14.3), org.Hs.eg.db package (v.3.10.0), and pathview package (v.1.26.0) based on the R language for GO and KEGG gene regulation pathways to evaluate the potential function of lncRNAs.

### Pan-Cancer Study of Key Prognostic-Related lncRNAs in PAAD Patients

#### Construct of Tissue Expression Pattern of lncRNA Genes

According lncRNA genes were identified from the Cox risk regression model. The normal human tissue gene expression data in the GTEx database and the phenotype file and key lncRNA expression levels in 33 tumor types were used to construct a healthy human body anatomical diagram of the target gene expression in the tissue. Among them, we categorize the organs and genders, and draw the medical anatomy diagrams of the expression of the target gene lncRNA in each organ. The Wilcox statistical test was used to calculate the differential expression of lncRNA genes in 33 cancers, and box plots were drawn based on the R language ggpubr package.

#### Pan-Cancer Survival Analysis

The median value of the lncRNA gene expression in each cancer type was used to divide cases into the high expression group and low expression group, and Kaplan–Meier curve analysis was performed to evaluate the survival between high and low expression groups. Survival analysis of pancreatic cancer patients was conducted for disease-related survival, disease-free interval and progression-free interval.

#### Correlation Analysis Between lncRNA Genes and Clinical Stage of Pan-Cancer

We used the non-parametric Kruskal–Wallis test to calculate the correlation between the expression of the lncRNA genes and the clinical stage in 33 cancers.

#### Analysis of the Correlation Between lncRNA Genes and Tumor Mutation Burden (TMB) in Pan-Cancer

Spearman correlation test was used to measure the correlation between the expression of the lncRNA gene and the mutation load of 33 tumors. We calculated the number of gene mutations in each tumor samples to obtain the tumor mutation load of each tumor sample. We set 0.3 < |r| < 0.5 for low correlation, 0.5 < |r| < 0.8 for medium correlation, and |r| > 0.8 for high correlation, with *P* ≤ 0.05. We used the R language fmsb package (version 0.7.0) to draw a radar chart of statistically significant cancer types.

#### Correlation Analysis Between lncRNA Genes and Microsatellite Instability (MSI)

We used Spearman’s method to analyze the correlation between the expression of the lncRNA genes and MSI in 33 tumors. MSI data were obtained from the results of the study by Bonneville et al. on pan-cancer MSI. This study calculated the MANTIS score for most tumor samples in TCGA database by calculating the distribution difference of the alleles of each microsatellite locus in the tumor-normal tissue paired sample and determining the average value as the MSI score value of the tumor-normal tissue paired sample.

#### Analysis of the Correlation Between lncRNA Genes and Pan-Cancer Tumor Microenvironment

Using the ESTIMATE algorithm, we estimated the content of stromal cells and immune cells in the tumor from tumor RNA-seq data. We analyzed and calculated the correlation between the expression of lncRNA genes in each cancer and the scores of ESTIMATE, and the correlation coefficient *r* > 0.4 and *P* < 0.001 were selected as the screening conditions.

#### Analysis of the Correlation Between lncRNA Genes and Pan-Cancer Immune Cell Infiltration

We used CIBERSORT to calculate the abundance of 22 types of infiltrating immune cells in tumor samples from 33 types of tumors in TCGA database. The correlation between the expression of the lncRNA genes and the 22 types of immune cells in each tumor was calculated based on the Spearman’s correlation analysis.

#### Analysis of the Correlation Between lncRNA Genes and Pan-Cancer Immune Genes

To examine the biological process and cell signal transduction pathways that the lncRNA genes may be involved in, we analyzed the relationship between the lncRNA genes and the immune genes based on Pearson’s correlation analysis and the immune gene set downloaded from the ImmPort database.

#### GSEA Function Enrichment Analysis of lncRNA Genes in Pan-Cancer

We downloaded the gene set file from the GSEA website and used the key lncRNA genes to GSEA to calculate the enrichment scores in the GO and KEGG pathways in 33 cancers.

## Results

### The 6-lncRNA Prognostic Risk Scoring Model for Pancreatic Cancer

**FIGURE 1:**
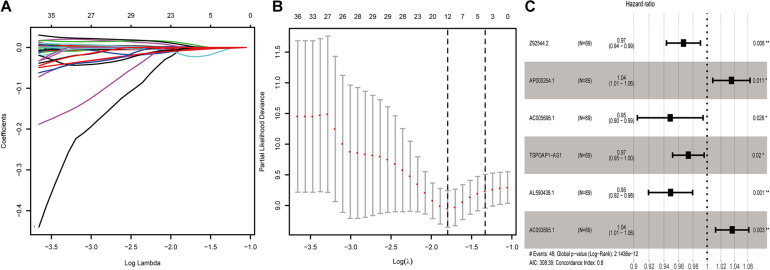
Screening of lncRNAs related to the prognosis of pancreatic cancer. **(A)** Results of lasso regression analysis of lncRNAs related to the prognosis of pancreatic cancer. **(B)** Cross-validation in lasso regression analysis of prognostic-related lncRNAs. **(C)** 6-lncRNA labeled risk ratio forest plot.

To examine potential lncRNAs related to the prognosis of pancreatic cancer patients, we used datasets from TCGA and GTEx databases as a combined dataset. We performed univariate Cox proportional hazard regression analysis for differential genes that analyzed from the combined dataset in the training data. A total of 112 lncRNAs that showed significant association with the prognosis of pancreatic cancer patients were obtained (*P* < 0.01). After lasso regression analysis, we deleted lncRNAs with high correlation or subordination candidates (Figures 1A,B), and 12 survival-related lncRNAs with high independence remained (*P* < 0.01) (Supplementary Table 1). Multi-factor Cox regression analysis based on the training dataset from the combined dataset screened out six prognostic lncRNAs. We constructed a prognostic risk scoring model based on the six survival-related lncRNAs.

Among the six lncRNAs, lncRNA AP005264.1, and lncRNA AC093895.1 showed a hazard ratio (HR) greater than 1.0, indicating these as risk factors. The HR of lncRNA AL590438.1, lncRNA TSPOAP1-AS1, lncRNA AC005696.1, and lncRNA Z92544.2 was less than 1.0, indicating these as protective factors (AIC: 308.39; C-index: 0.8) (Figure 1C).

### Construction of a Survival Prediction Model Based on the 6-lncRNA Model

**FIGURE 2:**
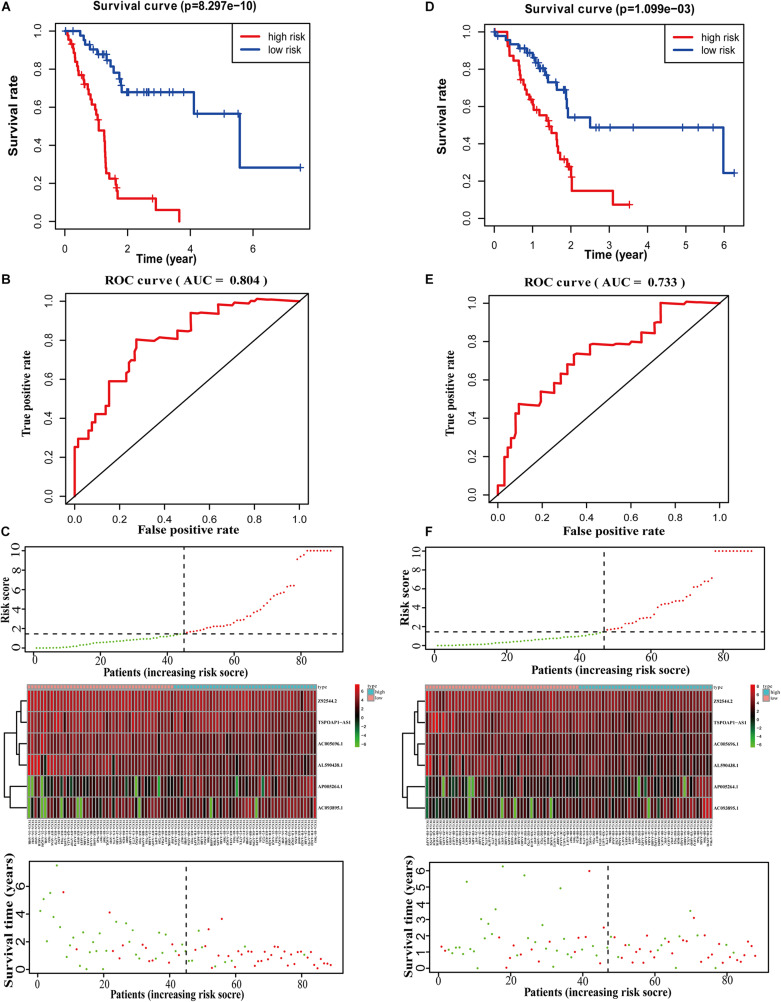
Performance evaluation of 6-lncRNA labeling in training set and test set. **(A)** Survival curve analysis of high-risk and low-risk groups. **(B)** 5-year ROC evaluation curve of 6-lncRNA labeling. **(C)** Patient risk score, survival status and lncRNA labeling expression profile. **(D)** KM analysis of the high and low risk groups in the test set. **(E)** 5-year ROC evaluation curve of 6-lncRNA marker in the test set. **(F)** Patient risk score, survival status and lncRNA marker expression profile in the test set.

We calculated scores for each pancreatic cancer patient in the training set according to the 6-lncRNA prognostic risk scoring model. Based on the median score of the patients in the training set (median = 3.85), we divided patients into the high-risk group (*n* = 44) and low-risk group (*n* = 45), a significant difference (*p*-value) between groups (*P* < 0.01). The average survival time of patients in the high-risk group was 1.03 years, which was significantly lower than the average survival time of patients in the low-risk group of 2.13 years (*P* < 0.001). Kaplan–Meier analysis demonstrated a significant difference in overall survival time (OS) between the two groups (*P* = 8.297E-10, log-rank test) (Figure 2A). The 3- and 5-year survival rates of the high-risk group were only 0.06 and 0%, while the 3- and 5-year survival rates of the low-risk group were 0.679 and 0.283%, respectively. At different time points, the survival rates of the high-risk and low-risk groups were relatively different (Supplementary Table 2). The area under the receiver operating characteristic (ROC) curve (AUC) of the 6-lncRNA model in the training group for 5 years reached 0.804 (Figure 2B), indicating that the prognostic risk scoring model predicted the 5-year survival rate of pancreatic cancer patients. The distribution of pancreatic cancer patient risk score, survival status and expression of the six lncRNAs in the high and low risk groups in the training set were shown in Figure 2C.

We next calculated the median score in the test set (*n* = 88) and divided the test dataset into a high-risk group (*n* = 42) and a low-risk group (*n* = 46). Kaplan–Meier analysis showed that there was a significant difference in the OS between the two groups (*P* = 1.099E-03, log-rank test) (Figure 2D), which is similar to the results in the training set. The 3- and 5-year survival rates of the high-risk group were only 0.07 and 0%, compared with 0.487 and 0.244% for the low-risk group. At different time points, the survival rates of the high-risk and low-risk groups were significant different (Supplementary Table 3). The 6-lncRNA the AUC of the model in the test set was 0.733 (Figure 2E). These results show that the prognostic risk scoring model is reliable in predicting the 5-year survival rate of pancreatic cancer patients in the test set. Figure 2F shows the risk score, survival time, number of deceased patients and the expression of the six lncRNAs in the high-risk group and the low-risk group.

### The 2-lncRNA Prognostic Risk Scoring Model for Pancreatic Cancer Patients and Survival Prediction Analysis

**FIGURE 3:**
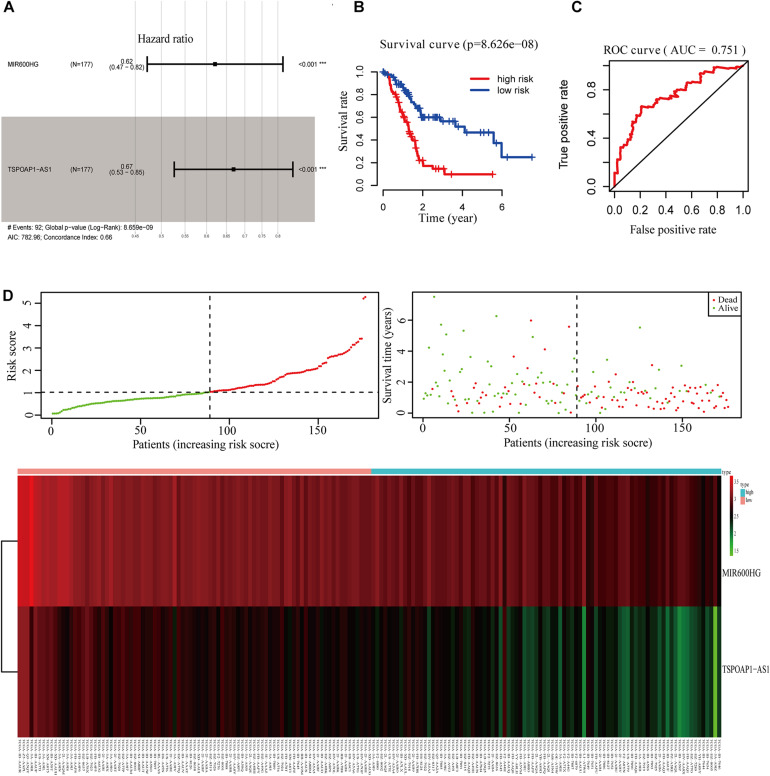
Survival analysis of 2-lncRNA. **(A)** 2-lncRNA labeled risk ratio forest plot. **(B)** KM survival curve of 2-lncRNAs high and low risk group. **(C)** ROC curve of 2-lncRNAs five-year survival rate. **(D)** Comparison of 2-lncRNA high and low risk groups.

We performed survival analysis on the overall dataset of TCGA pancreatic cancer patients without the GTEx dataset. A total of 46 lncRNAs were significantly related to the OS of pancreatic cancer patients (*P* < 0.05). After univariate Cox proportional hazard regression analysis, we obtained 7 lncRNAs, which were entered into the multivariate Cox ratio risk regression analysis (Supplementary Table 4). Finally, we obtained a prognostic risk score model for pancreatic cancer patients with two lncRNAs. The HR of lncRNA MIR600HG and TSPOAP1—AS1 were less than 1.0, indicating these as protective factors in the prognosis of pancreatic cancer (AIC: 782.96; C-index: 0.66) (Figure 3A).

Using the median value of the 2-lncRNA prognostic risk scoring model, TCGA pancreatic cancer patient samples were divided into high-risk and low-risk groups. Kaplan–Meier analysis showed that there was a significant difference in the OS between the high-risk and low-risk groups (*P* < 0.001) (Figure 3B). Moreover, AUC of the model in 5 years was 0.751 (Figure 3C), indicating that the 2-lncRNA marker had a reliable performance in predicting the 5-year survival rate of pancreatic cancer patients in the test set. Between the high-risk and low-risk groups, the two lncRNAs in the prognostic risk model were sorted and compared in terms of risk score, overall survival time and survival status (Figure 3D).

### Genes Correlated With Key lncRNAs in the Prognostic Risk Scoring Model

From all gene expression profiles of pancreatic cancer, 19,658 PCGs and 14,142 non-protein coding lncRNA genes were obtained. After standardization, we obtained 16,988 PCGs. Among the lncRNAs from the two models, lncRNA TSPOAP1-AS1 and lncRNA MIR600HG genes were selected among all candidates. For these two key lncRNA genes, Pearson correlation analysis was performed one by one with the normalized PCG expression dataset of pancreatic cancer patients. A total of 1673 PCGs related to TSPOAP1-AS1 and 2,172 CGs related to MIR600HG were obtained (Supplementary Table 6).

**FIGURE 4:**
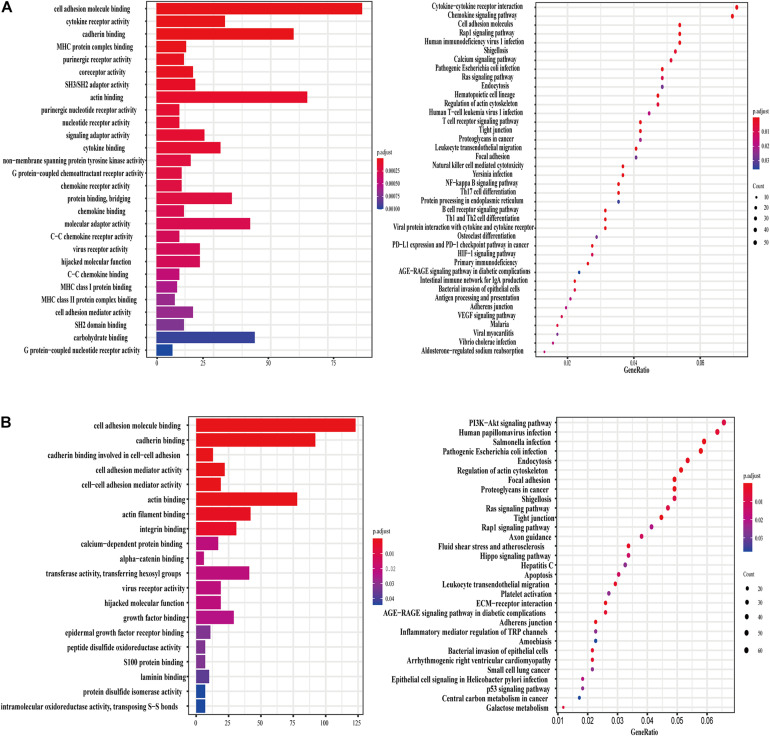
lncRNA TSPOAP1-AS1 and MIR600HG related genes and functional enrichment. **(A)** GO enrichment and KEGG pathway enrichment of lncRNA TSPOAP1-AS1 related genes. **(B)** GO enrichment and KEGG pathway enrichment of lncRNA MIR600HG related genes.

PCGs related to lncRNA TSPOAP1-AS1 were enriched in 27 GO terms that were mostly involved in seven functions, including extracellular matrix, protein bridging, human major histocompatibility system MHC-II, G protein-coupled receptor-mediated signaling pathways and other functions. KEGG pathway analysis showed that PCGs were mainly enriched in five pathways including immune cell activation, anti-inflammatory response, JAK-STAT signaling pathway, protein secretion and glycogen synthesis (Figure 4A). The PCGs of lncRNA MIR600HG showed 28 enriched GO terms, mainly focused on five biological processes and functions: calcium ion-dependent cell adhesion, purine nucleoside metabolism, uridine cyclase activation, GTP hydrolase activation and binding to epidermal growth factor receptor. The KEGG pathways were related to cytoskeleton adjustment, leukocyte migration, cancer tissue proteoglycan regulation and bacterial invasion of endothelial cells (Figure 4B).

The two lncRNA genes were also involved in a variety of cancer-related pathways in pan-cancer. We used GSEA analysis and found that in Cholangiocarcinoma (CHOL), TSPOAP1-AS1 is involved in biological processes such as lymphocyte activation, B cell activation, immune cell signal transduction receptor regulation and immune effect response regulation, while MIR600HG is involved cell signaling pathways such as methylated CpG protein regulation, DNA transcription factor binding and RNA polymerase binding, and lncRNA MIR600HG negatively regulates these functional pathways. Participate in gene silencing in DLBC, gene silencing under the action of non-coding RNA. Participate in gene silencing and non-coding RNA in GBM (Supplementary Figures 1, 2).

### Analysis of lncRNA Genes in Non-cancerous Tissues and Pan-Cancer

**FIGURE 5:**
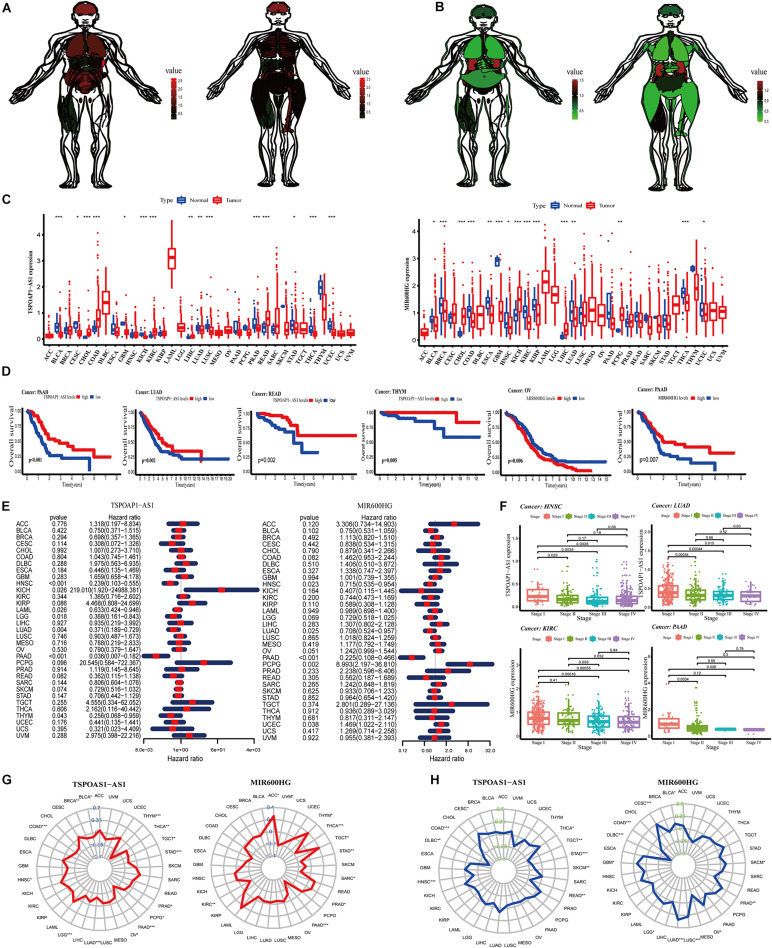
Analysis of target genes in normal human organs and pan-cancer. **(A)** Expression of lncRNA TSPOAP1-AS1 in Male and female normal human organs. **(B)** Expression of lncRNA MIR600HG in Male and female normal human organs. **(C)** Difference in expression of TSPOAP1-AS1 and MIR600HG in 33 tumors. **(D)** Survival curve analysis of lncRNA TSPOAP1-AS1 and MIR600HG in multiple cancer patients. **(E)** lncRNA TSPOAP1-AS1 and MIR600HG risk ratio forest diagram. **(F)** The correlation between TSPOAP1-AS1 and MIR600HG and clinical stages in various cancers. **(G)** Correlation between TSPOAP1-AS1 and MIR600HG and pan-cancer TMB. The blue value is the scale of the correlation coefficient, “*,” “**,” and “***,” respectively, represent: *P* < 0.05, *P* < 0.01, *P* < 0.001. **(H)** Correlation between TSPOAP1-AS1, MIR600HG and pan-cancer MSI. The green value is the scale of correlation coefficient, “*,” “**,” and “***,” respectively, represent: *P* < 0.05, *P* < 0.01, *P* < 0.001.

Genes show differences in expression in tissues and between different sexes. We used the expression levels of the lncRNA TSPOAP1-AS1 and MIR600HG genes in each organ to draw the medical anatomy of normal human organs of both sexes. In general, the two lncRNAs gene expression were expressed at low levels in all examined tissues. LncRNA TSPOAP1-AS1 had the highest expression in the spleen and high expression in the brain and lung tissues. The intestinal system in men and the long bones in women had higher expression levels of lncRNA TSPOAP1-AS1. MIR600HG showed relatively high expression in kidney tissues and low expression levels in lung tissues, intestines of males and skeletal muscles of females (Figures 5A,B).

We next examined gene expressions of the two lncRNAs in tumor tissues and normal tissues in 33 types of tumors (Supplementary Table 5). LncRNA TSPOAP1-AS1 was significantly higher or lower in bladder cancer (BLCA) and 10 types of cancer (*P* < 0.001). TSPOAP1-AS1 also showed significant differences (*P* < 0.01 or *P* < 0.05) in expression in cervical cancer (CESC) and lung adenocarcinoma (LUDA). We also detected significantly different expression of lncRNA MIR600HG in 10 cancers including breast cancer (BRCA) and colon cancer (COAD) (Figure 5C).

We divided patients into high- and low-expression groups according to the median expression of lncRNA TSPOAP1-AS1KM and MIR600HG and examined OS in the 33 cancers. Significant differences in OS between high- and low-expression groups were observed in PAAD, LUAD, READ, and THYM, among other cancers, with the most significant difference in PAAD. There was a significant prognostic difference between OV and PAAD patients in low and high expression of lncRNA MIR600HG groups (*P* < 0.01) (Figure 5D and Supplementary Figure 2A). Cox proportional hazard regression analysis was performed in 33 cancers using gene expression of the lncRNAs. LncRNA TSPOAP1-AS1 had a significant relationship with the prognosis of HNSC and PAAD patients (*P* < 0.001) and was a low-risk factor for prognosis. LncRNA MIR600HG had a significant correlation with the prognosis of PAAD patients (*P* < 0.001) and was also a low-risk factor (Figure 5E).

Significant differences in disease-related survival between high- and low-expression groups were detected in a variety of cancers (*P* < 0.05), with high significance observed with PAAD (Supplementary Figure 3B). The lncRNA TSPOAP1-AS1 was a high-risk factor for prognosis in KICH and a low-risk factor in other cancer types. The lncRNA MIR600HG was a high-risk factor for prognosis in PCPG and a low-risk factor for other cancers (Supplementary Figure 3C). A significant difference was detected in disease-free interval according to lncRNA TSPOAP1-AS1 expression in COAD, and lncRNA TSPOAP1-AS1 expression in COAD and KIRP both had a significant level of difference, and were high-risk factors for patient prognosis in the two cancers. A significant differences between the high and low lncRNA MIR600HG risk groups were observed in LUSC, PAAD, and PRAD, and a significant prognosis difference in many cancers such as PAAD, among which lncRNA MIR600HG were low risk factors in PAAD and THCA (Supplementary Figure 3D). In the examination of progression-free interval, both the high and low risk groups showed significant survival differences in a variety of cancers. The high and low risk groups of the two lncRNAs showed significant differences in survival in PAAD. LncRNA TSPOAP1-AS1 was a significant prognostic factor in a variety of cancers, among which TSPOAP1-AS1 was a high risk factor in UVM. LncRNA MIR600HG was a significant prognostic factor in many cancers such as PAAD and PRA, among which MIR600HG was a low risk factor in PAAD (Supplementary Figure 3E).

We next performed non-parametric Kruskal test analysis on the correlation between the two lncRNAs and the clinical stage of the patients in 33 cancer types. The expression of lncRNA TSPOAP1-AS1 in HNSC, KIRP, LIHC, LUAD, SKCM, TGCT, and THCA showed significant differences in clinical stages, among them, HNSC and LUAD had the most significant performance. The expression of lncRNA TSPOAP1-AS1 showed a significant difference between clinical stage I and II clinical pancreatic cancer samples (*P* < 0.05) and a greater significant difference between stage III and IV clinical pancreatic cancer samples in HNSC (*P* < 0.001). In LUAD, there was also a highly significant difference between stage I clinical pancreatic cancer samples and stages II and III clinical pancreatic cancer samples (*P* < 0.001), and stages I and IV clinical pancreatic cancer samples had a significant difference (*P* < 0.05) (Figure 5F and Supplementary Figure 4). A statistically significant correlation was observed between lncRNA MIR600HG and clinical stage in KIRC, PAAD and 10 kinds of cancers; the expression of MIR600HG was highly statistically significant in clinical stage III clinical pancreatic cancer samples and IV clinical pancreatic cancer samples in KIRC (*P* < 0.001).

We obtained the total number of mutations to obtain the TMB and calculated the Spearman correlation coefficient between TSPOAP1-AS1 and MIR600HG gene expression in each tumor and TMB. We found that lncRNA TSPOAP1-AS1 showed a negative correlation with TMB in THYM (*r* = −0.626) and PAAD (*r* = −0.435), and MIR600HG showed a low negative correlation with TMB in PAAD (*r* = −0.320) (Figure 5G). We also performed Spearman’s correlation analysis between the lncRNAs and MSI. The lncRNA TSPOAP1-AS1 had a negative or no correlation with MSI of most cancer types, while lncRNA MIR600HG had a higher correlation with MSI in more tumors (Figure 5H).

### Correlation Analysis of lncRNA Genes and Tumor Immunity in Pan-Cancer

**FIGURE 6:**
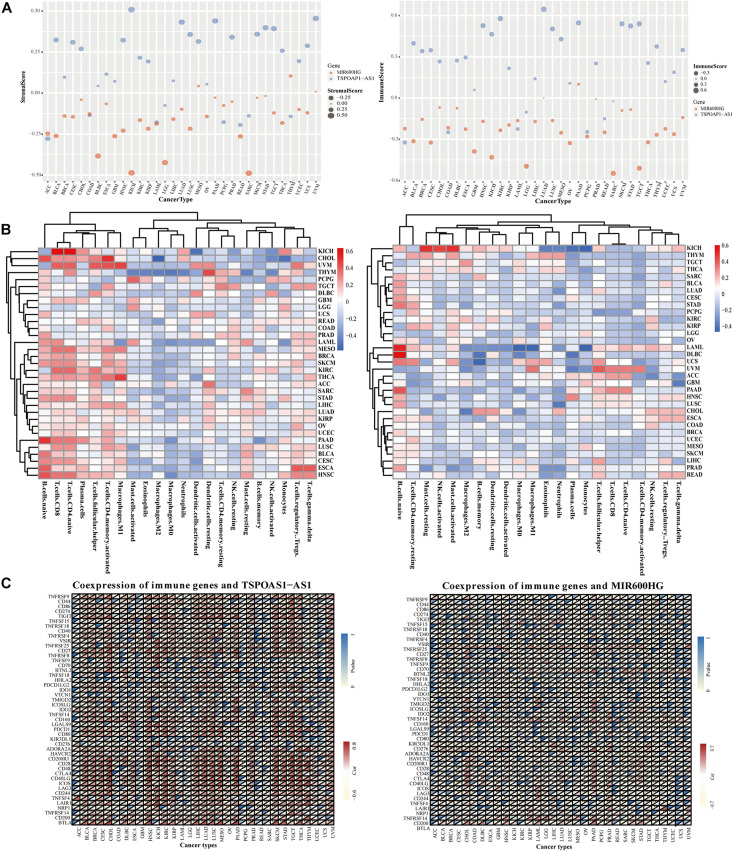
Correlation analysis of target genes and tumor immunity in pan cancer. **(A)** Correlation of lncRNA TSPOAP1-AS1, MIR600HG and the content of immune cells and stromal cells in pan-cancer. **(B)** The correlation between the expression of lncRNA TSPOAP1-AS1, MIR600HG and the infiltration of multiple cancer immune cells. **(C)** Results of co-expression of TSPOAP1-AS1, MIR600HG and immune genes in 33 tumors. Blue represents *P*-value and Red represents correlation coefficient.

We next analyzed the relationship between the expression of lncRNA TSPOAP1-AS1 and MIR600HG and the levels of immune cells and stromal cells of 33 tumors. LncRNA TSPOAP1-AS1 showed a statistically markedly positive correlation with immune cell content and stromal cell content in KICH, LUAD, and STAD. TSPOAP1-AS1 showed a significant positive correlation with immune cell content in BLCA, HNSC, KIRC, LUSC, MESO, SKCM, and TGCT while it only showed a positive correlation with stromal cells in in UVM. LncRNA MIR600HG had a significant correlation with immune cells and stromal cell content in KICH, LGG, and SARC, but it had significant correlation with immune cells only in TGCT (|r| > 0.4, *P* < 0.001) (Figure 6A and Supplementary Figure 5).

The gene expression of lncRNA TSPOAP1-AS1 was significantly correlated with the infiltration ratio of 10 kinds of immune cells in seven cancers (*r* > 0.4, *P* < 0.001). Such as ESCA, it was related to naive B cells and regulatory T cells (Supplementary Figure 6). There was also a significant negative correlation of lncRNA TSPOAP1-AS1 with the content of memory B cells in PAAD, while the expression of lncRNA MIR600HG was significantly negatively correlated with the content of initial B cells in DLBC, LAML, and PAAD (*r* < −0.4, *P* < 0.001) and significantly positively correlated with the content of monocytes in LAML (*r* > 0.4, *P* < 0.001) (Figure 6B). LncRNA TSPOAP1-AS1 had immune genes (0.5 < |*r*| < 0.8) that were more than moderately related to it in 23 kinds of tumors, which were mainly distributed in, for example, PAAD (17 immune-related genes). LncRNA TSPOAP1-AS1 showed a significant correlation with the immune-related gene *BTLA* in 15 kinds of cancers, with a strong correlation in PAAD and CHOL (|*r*| > 0.8), and the other mainly significant immune genes included CD28 (in 18 types of cancer) and CD40LG (in 14 types of cancer) (Figure 6C). LncRNA MIR600HG showed a significant correlation with several immune genes in only eight tumors (CHOL, DLBC, GBD, LAML, LGG, PAAD, SARC, and THYM); This lncRNA was associated with fewer immune genes compared with TSPOAP1-AS1 and the degree of correlation was low.

## Discussion

Pancreatic cancer is characterized by high malignancy, rapid development, and strong invasiveness, and the prognosis of pancreatic cancer is very poor. The selection of pancreatic cancer cases suitable for surgical resection is strict, requiring no distant metastasis, superior mesenteric vein structure, uninvaded superior mesenteric artery, and normal peripheral space. Only 12–14% of patients are suitable for surgery, and the surgery is complicated. The surgical resection rate is only 40%, and surgery is associated with multiple postoperative complications. Notably, increasing the operation rate and expanding the scope of surgical resection have not improved patient prognosis. Therefore, early diagnosis and accurate prognosis of pancreatic cancer are very important.

The identification of pancreatic cancer–specific markers is critical but remains challenging. CA19-9 is widely used as a biomarker in patients with pancreatic cancer in clinical practice. It has relatively high sensitivity and good specificity for pancreatic cancer, with a positivity rate between 8 and 95%, and it decreases with the improvement of the condition after surgery (Liu et al., 2020). Some studies have suggested that CA19-9 has great value in predicting the prognosis of patients with pancreatic cancer (Humphris et al., 2012; Nakamura et al., 2019). However, CA19-9 also has a false negative rate in pancreatic cancer patients. CA19-9 is not expressed in the Lewis blood group population, which means that the serum CA19-9 level of these pancreatic cancer patients is not elevated (He et al., 2014; Ren, 2020). In patients with early pancreatic cancer (tumor < 3 cm), the sensitivity of CA19-9 detection is low, often less than 50% (Gruber and Zavolan, 2019). In terms of protein molecular markers, the combination of macrophage inhibitory factor (MIC-1) and alcohol dehydrogenase (ADH) has been used in the diagnosis of early pancreatic cancer patients, with a specificity and sensitivity reaching 94 and 45.8%, respectively. ADH alone can reach 83.3 and 62% specificity and sensitivity (Sun et al., 2019). MicroRNA biomarkers have also been widely studied in the diagnosis, pre- and post-operative evaluation and prognosis prediction of pancreatic cancer. A recent study of 372 patient samples based on biochips showed that miRNA-7 has great potential in predicting the survival of pancreatic cancer patients (Ye et al., 2014). In one study, a meta-analysis was used to screen several serum miRNAs that can indicate the prognosis of patients with pancreatic cancer (Chen et al., 2019). However, these miRNAs often need to be used in combination with other biomolecular examinations for screening. The process is cumbersome and the results are not satisfactory. Therefore, identifying new specific and sensitive biomarkers for pancreatic cancer patients is necessary.

LncRNAs have been highly valued in recent years as biomarkers, but the functions and mechanisms of action of most lncRNAs have not been fully elucidated (Spodzieja et al., 2017). Recently, 42 lncRNAs with clinical biomarker potential have been reported, and 3 lncRNAs with important prognostic significance have been identified and screened. Several studies have reported good prognostic models for pancreatic cancer, but these reports used pancreatic cancer data from TCGA and did not have equal numbers of positive and negative samples during data collection. Considering the lack of non-cancerous samples in the pancreatic cancer section of the TCGA database, here we introduced non-tumor pancreatic tissue samples from the GTEx database to construct an ideal pancreatic cancer prognosis prediction model.

In this study, we obtained two risk prognostic models of lncRNAs. The 2-lncRNA model was used for comparison to evaluate the advantages and disadvantages of the 6-lncRNA model. The working performance of the 6-lncRNA model was better than that of the 2-lncRNA model, indicating that the pancreatic tissue samples we introduced from the GTEx database were necessary. However, because the 2-lncRNA model contains only two lncRNAs, clinical practice may be convenient. We propose that the 2-lncRNA model is used to first screen surgical patients, and if the results are judged as short-lived, then the 6-lncRNA model risk score can be used to determine prognosis. We also selected the two most statistically significant lncRNAs, TSPOAP1-AS1 and MIR600HG, from the models as key genes for correlation research. Both lncRNAs were effective in predicting the prognosis of pancreatic cancer patients and showed a certain correlation with the tumor microenvironment. Both lncRNA genes showed a significant correlation with clinical staging in lung adenocarcinoma (LUAD). LncRNA TSPOAP1-AS1 is also a potential prognostic biomarker for HNSC in addition to PAAD. TSPOAP1-AS1 is a low-risk factor for ESCA patients and a high-risk factor for KICH patients. According to the phenomenon, we speculated that the content of CD8+ T cells in KICH patients may not be an independent prognosis risk factors. Notably, in two pathological types of kidney cancer (renal clear cell carcinoma and renal papillary cell carcinoma), MIR600HG showed a highly significant correlation with clinical stage, suggesting that two lncRNAs may promote tumor cell proliferation or de-inhibition in related tumors. The lncRNA MIR600HG had a significant negative correlation with the initial B cell content in diffuse large B-cell lymphoma (DLBC), acute myeloid leukemia (LAML) and pancreatic cancer (PAAD).

Our results suggest that TSPOAP1-AS1 is mainly involved in the regulation of cellular immune response, while MIR600HG is involved in the regulation of substance metabolism and regulation of cell signal transduction. This further illustrates the important roles of lncRNA TSPOAP1-AS1 and MIR600HG in the occurrence and development of various tumors. In conclusion, here we obtained two prognostic models of lncRNAs by combining two datasets, and our results suggest that lncRNAs TSPOAP1-AS1 and MIR600HG play important roles in the development and diagnosis of multiple cancers.

## Data Availability Statement

Publicly available datasets were analyzed in this study. The names of the repository/repositories and accession number(s) can be found in the article/Supplementary Material.

## Author Contributions

XT and MZ conceived the whole study and performed all data acquisition, programming, and code execution. SW, YZ, and YD designed and performed the experiments. LS and FX carried out the sample collection. FX and XP carried out the data analysis. MZ wrote the manuscript. All authors have read and approved the final manuscript and contributed to the work presented in this manuscript.

## Conflict of Interest

The authors declare that the research was conducted in the absence of any commercial or financial relationships that could be construed as a potential conflict of interest.

## Footnotes

1 https://portal.gdc.cancer.gov/

2 https://gtexportal.org

3 https://xenabrower.net/datapages

4 ftp://ftp.ensembl.org/pub/current_gtf

5 https://www.immport.org
