# Supplementary material for: The Biological Function Delineated Across Pan-Cancer Levels Through lncRNA-Based Prognostic Risk Assessment Factors for Pancreatic Cancer
Source: Front Cell Dev Biol. 2021 Jun 14;9:694652. doi: 10.3389/fcell.2021.694652 (PMC8236889; doi:10.3389/fcell.2021.694652)

Supplementary Material

Supplementary Table 1. 12 overall survival time-related lncRNAs in pancreatic cancer patients

Ensemble ID	Gene name	Chromosome （GRCh38）	Hazad ratio	HR（95%CI）	*p*-value
ENSG00000279441	Z92544.2	Chr16: 678,645-679,061	0.96	0.94-0.99	6.48E-03
ENSG00000233818	AP000695.2	Chr21: 36,445,731-36,532,408	1.01	1.00-1.02	2.6E-04
ENSG00000261420	AL022069.1	Chr6: 166,383,189-166,384,824	0.80	0.70-0.91	7.61E-04
ENSG00000240731	AL139287.1	Chr1: 1,317,581-1,318,689	0.98	0.97-0.99	1.73E-04
ENSG00000267069	AP005264.1	Chr18: 12,288,308-12,291,488	1.03	1.01-1.06	1.07E-02
ENSG00000236723	AL606760.2	Chr1: 53,209,783-53,213,775	0.88	0.82-0.95	3.19E-04
ENSG00000262050	AC005696.1	Chr17: 2,712,309-2,712,833	0.95	0.95-0.99	2.79E-02
ENSG000002651	TSPOAP1-AS1	Chr17:			

48		5,832,450-58,353,727	0.97	0.95-0.99	2.0E-02
ENSG00000272871	AL159169.2	Chr9: 14,588,797-14,590,065	0.88	0.83-0.95	3.19E-04
ENSG00000250410	AC112722.1	Chr4: 185,370,725-185,390,928	0.83	0.74-0.94	2.25E-03
ENSG00000272235	AL590438.1	Chr1: 3,306,636-3,310,096	0.95	0.92-0.98	1.16E-03
ENSG00000200024	AC093895.1	Chr4: 87,743,952-87,744,053	1.04	1.01-1.06	3.08E-03

Supplementary Table 2. Survival rates of the high-risk group and the low-risk group at different time points in the training set

	Time(year)	Number of risks	Survival rate	Standard error	Low 95%CI	High 95%CI
High risk group	0.6137	31	0.7207	0.0686	0.5981	0.868
	1.0000	22	0.5605	0.0785	0.4259	0.738
	1.4164	9	0.2248	0.0691	0.1231	0.410
	1.6822	4	0.1204	0.0584	0.0466	0.311
	2.9014	2	0.0602	0.0516	0.0112	0.323
Low risk group	1.036	35	0.878	0.0513	0.7826	0.984
	1.556	25	0.781	0.0697	0.6560	0.930
	1.811	20	0.679	0.0819	0.5359	0.860
	4.115	6	0.566	0.1238	0.3684	0.869
	5.578	2	0.283	0.2094	0.0663	1.000

Supplementary Table 3. Survival rates of the high-risk group and the low-risk group at different time points in the test set

	Time(year)	Number of risks	Survival rate	Standard error	Low 95%CI	High 95%CI
High risk group	0.638	33	0.8205	0.0615	0.7085	0.950
	1.003	23	0.6097	0.0790	0.4730	0.786
	1.493	14	0.4577	0.0840	0.3195	0.656
	2.005	5	0.2218	0.0817	0.1078	0.457
	3.096	2	0.0739	0.0662	0.0128	0.428
Low risk group	1.0027	34	0.861	0.0527	0.7641	0.971
	1.4000	20	0.730	0.0761	0.5949	0.895
	1.9233	12	0.542	0.0993	0.3789	0.776
	2.5014	10	0.487	0.1031	0.3220	0.738
	5.9781	2	0.244	0.1799	0.0574	1.000

Supplementary Table 4. survival-related lncRNAs in pancreatic cancer patients

Ensemble ID	Gene name	Chromosome （GRCh38）	Hazad ratio	Z-value	*p*-value
ENSG00000265148	TSPOAP1-AS1	Chr17: 5,832,450-58, 353,727	0.57675547	-5.042947509	4.58E-07
ENSG00000236901	MIR600HG	Chr 9: 123,109,494-123, 115,477	0.536490228	-4.848993182	1.24E-06
ENSG00000180539	C9orf139	Chr 9: 137,027,464-137,037,957	0.626592287	-4.286347527	1.82E-05
ENSG00000274712	AC005332.4	Chr 17: 68205489-68207493	0.593293749	-4.139493901	3.48E-05

ENSG00000254420	AP003086.1	Chr 11: 78,324,758-78,444,049	0.720749482	-3.563364984	0.000366131
ENSG00000248335	AC096733.2	Chr 4: 140,712,168-140,716,081	0.726354065	-3.489513305	0.000483901
ENSG00000261490	AC005674.2	Chr 4: 10,068,089-10,073,019	0.73652538	-3.316455384	0.000911671

Supplementary Table 5. Cancer abbreviations table.

Cancer abbreviations	Full name of cancer in English
ACC	Adrenocortical carcinoma
BLCA	Bladder Urothelial Carcinoma
BRCA	Breast invasive carcinoma
CESC	Cervical squamous cell carcinoma and endocervical adenocarcinoma
CHOL	Cholangiocarcinoma
COAD	Colon adenocarcinoma
DLBC	Lymphoid Neoplasm Diffuse Large B-cell Lymphoma
ESCA	Esophageal carcinoma
GBM	Glioblastoma multiforme
GBMLGG	Glioma
HNSC	Head and Neck squamous cell carcinoma
KICH	Kidney Chromophobe

KIPAN	Pan-kidney cohort （KICH+KIRC+KIRP）
KIRC	Kidney renal clear cell carcinoma
KIRP	Kidney renal papillary cell carcinoma
LAML	Acute Myeloid Leukemia
LGG	Brain Lower Grade Glioma
LIHC	Liver hepatocellular carcinoma
LUAD	Lung adenocarcinoma
LUSC	Lung squamous cell carcinoma
MESO	Mesothelioma
OV	Ovarian serous cystadenocarcinoma
PAAD	Pancreatic adenocarcinoma
PCPG	Pheochromocytoma and Paraganglioma
PRAD	Prostate adenocarcinoma
READ	Rectum adenocarcinoma
SARC	Sarcoma
SKCM	Skin Cutaneous Melanoma
STAD	Stomach adenocarcinoma
TGCT	Testicular Germ Cell Tumors
THCA	Thyroid carcinoma
THYM	Thymoma
UCEC	Uterine Corpus Endometrial Carcinoma
UCS	Uterine Carcinosarcoma
UVM	Uveal Melanoma

Supplementary Figure 1. **GSEA analysis of lncRNA TSPOAP1-AS1 in a variety of cancers.**

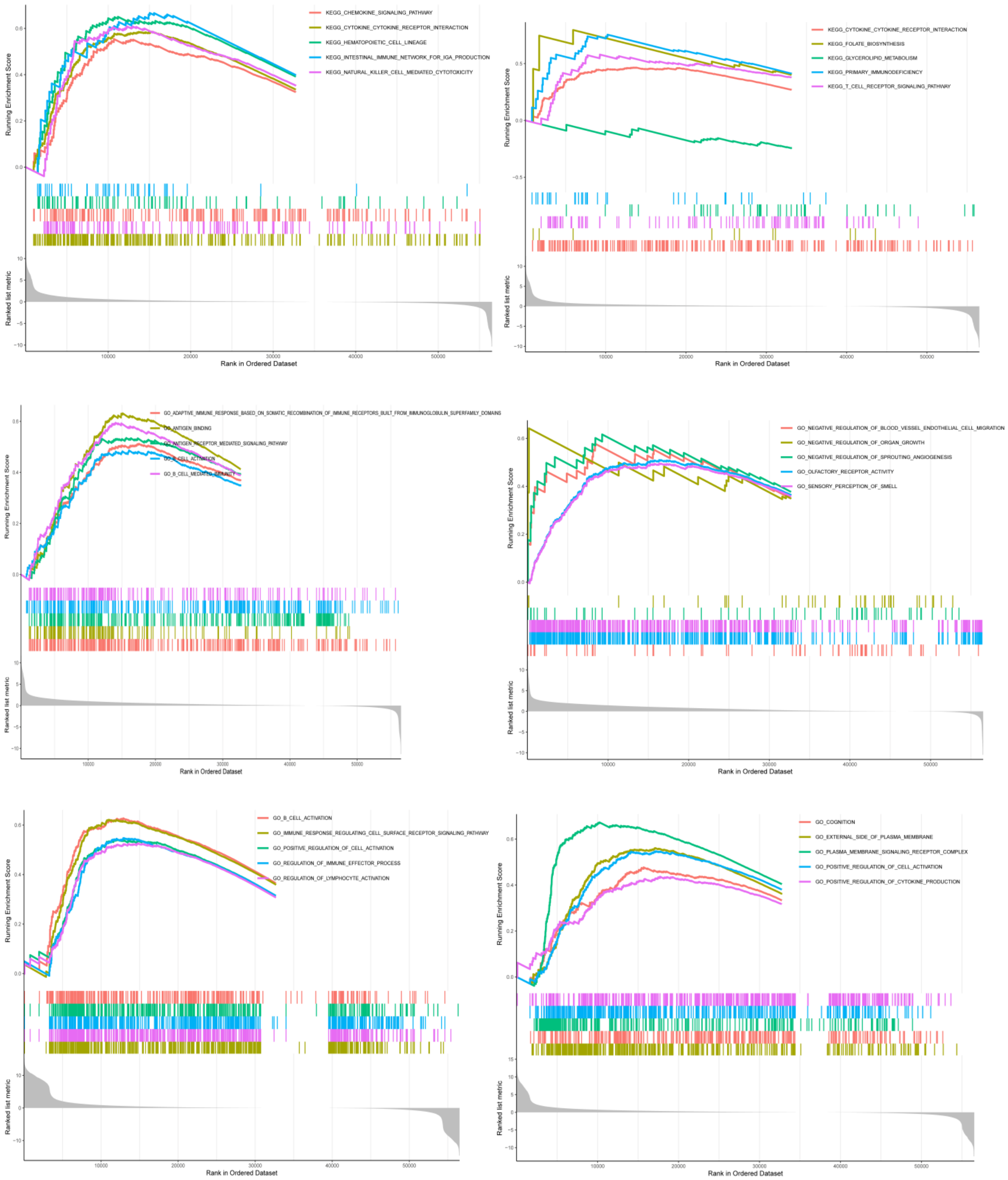

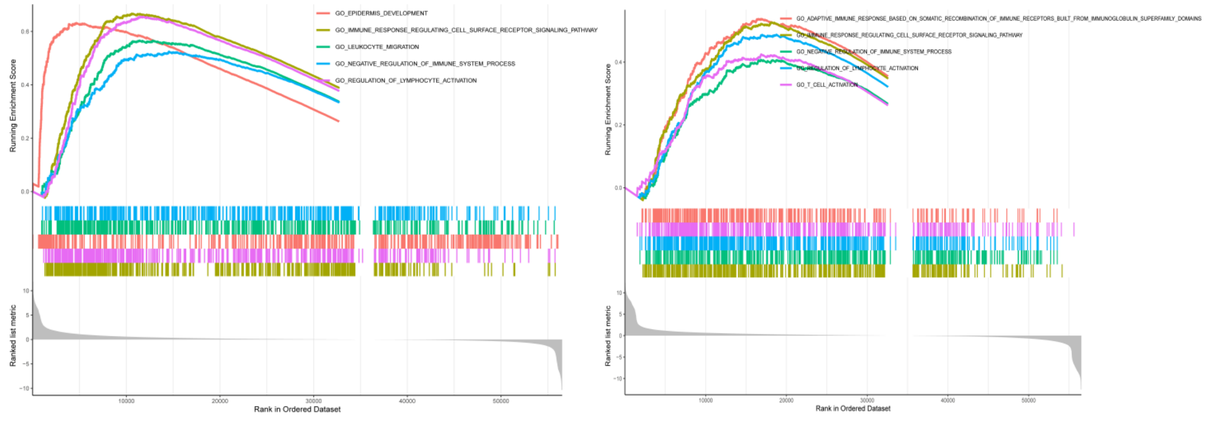

Supplementary Figure 2. **GSEA analysis of lncRNA MIR600HG in a variety of cancers.**

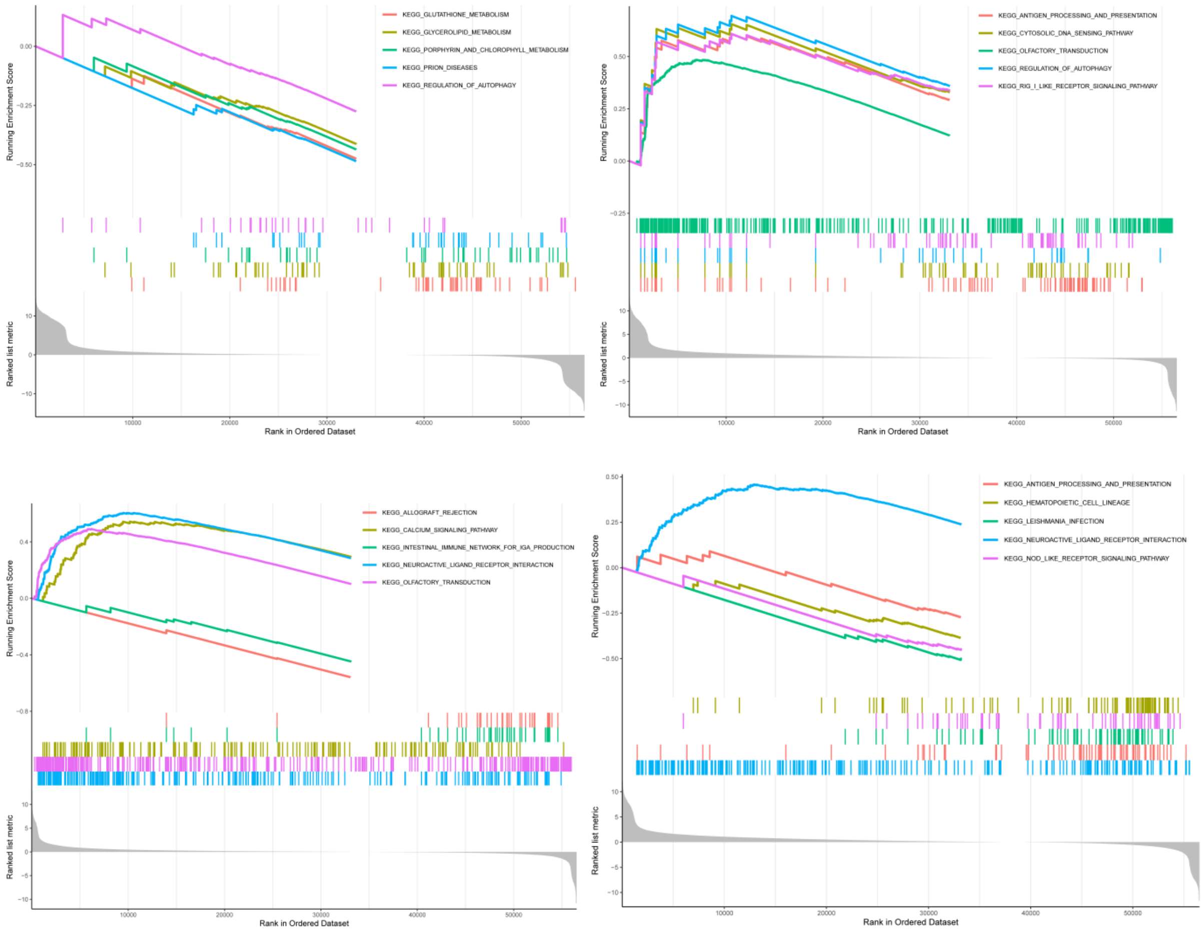

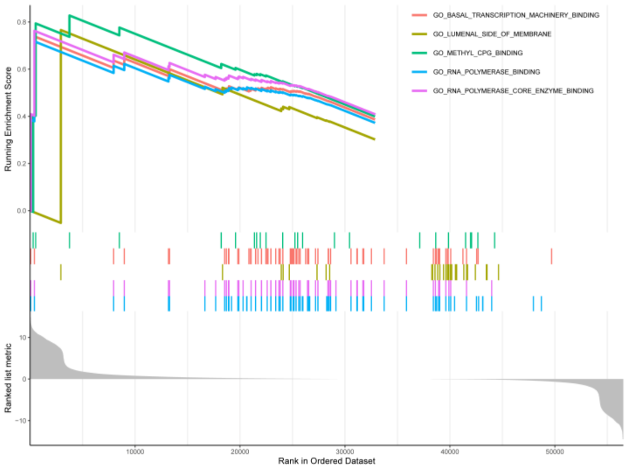
GO_BASAL_TRANSCRIPTION_MACHINERY_BINDING
GO_LUMENAL_SIDE_OF_MEMBRANE
GO_METHYL_CPG_BINDING
GO_RNA_POLYMERASE_BINDING
GO_RNA_POLYMERASE_CORE_ENZYME_BINDING
Running Enrichment Score
Ranked list metric
Rank in Ordered Dataset

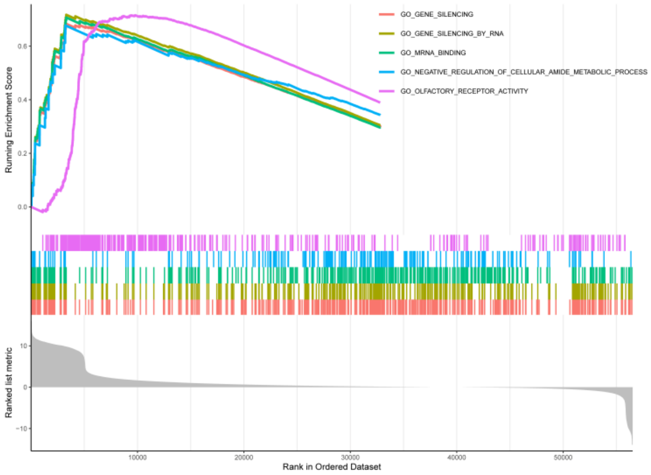
GO_GENE_SILENCING
GO_GENE_SILENCING_BY_RNA
GO_MRNA_BINDING
GO_NEGATIVE_REGULATION_OF_CELLULAR_AMIDE_METABOLIC_PROCESS
GO_OLFACTORY_RECEPTOR_ACTIVITY
Running Enrichment Score
Ranked list metric
Rank in Ordered Dataset

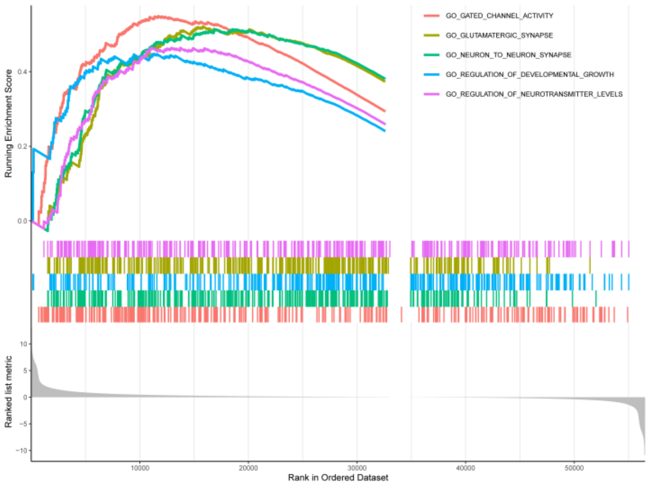
GO_GATED_CHANNEL_ACTIVITY
GO_GLUTAMATERGIC_SYNAPSE
GO_NEURON_TO_NEURON_SYNAPSE
GO_REGULATION_OF_DEVELOPMENTAL_GROWTH
GO_REGULATION_OF_NEUROTRANSMITTER_LEVELS
Running Enrichment Score
Ranked list metric
Rank in Ordered Dataset

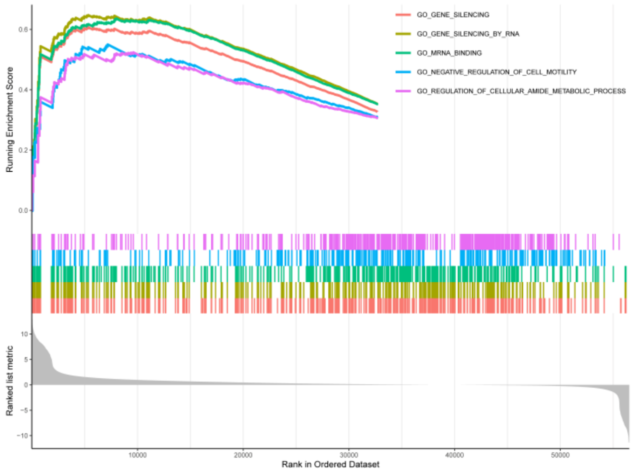
GO_GENE_SILENCING
GO_GENE_SILENCING_BY_RNA
GO_MRNA_BINDING
GO_NEGATIVE_REGULATION_OF_CELL_MOTILITY
GO_REGULATION_OF_CELLULAR_AMIDE_METABOLIC_PROCESS
Running Enrichment Score
Ranked list metric
Rank in Ordered Dataset

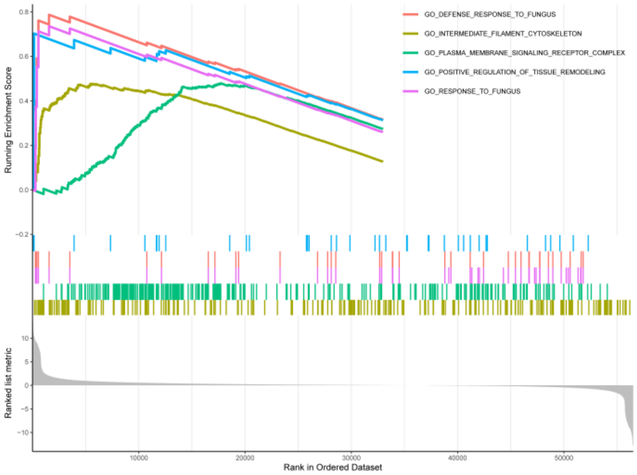
GO_DEFENSE_RESPONSE_TO_FUNGUS
GO_INTERMEDIATE_FILAMENT_CYTOSKELETON
GO_PLASMA_MEMBRANE_SIGNALING_RECEPTOR_COMPLEX
GO_POSITIVE_REGULATION_OF_TISSUE_REMODELING
GO_RESPONSE_TO_FUNGUS
Running Enrichment Score
Ranked list metric
Rank in Ordered Dataset

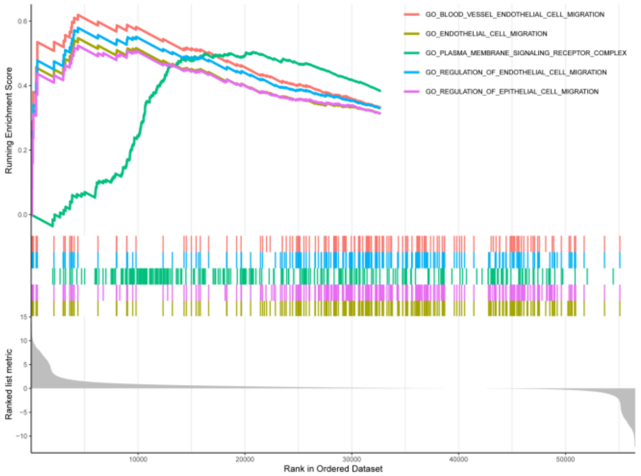
GO_BLOOD_VESSEL_ENDOTHELIAL_CELL_MIGRATION
GO_ENDOTHELIAL_CELL_MIGRATION
GO_PLASMA_MEMBRANE_SIGNALING_RECEPTOR_COMPLEX
GO_REGULATION_OF_ENDOTHELIAL_CELL_MIGRATION
GO_REGULATION_OF_EPITHELIAL_CELL_MIGRATION
Running Enrichment Score
Ranked list metric
Rank in Ordered Dataset

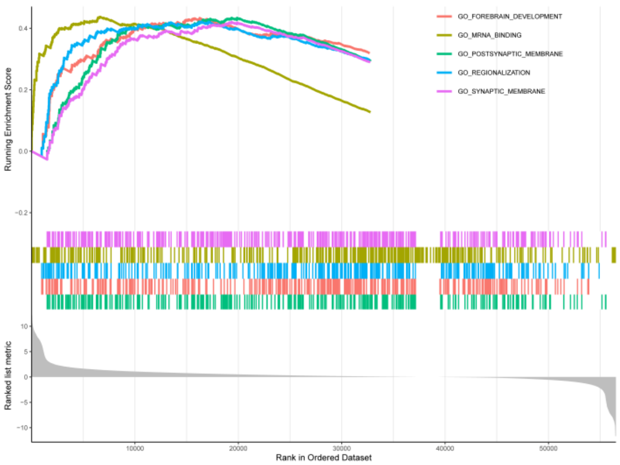
GO_FOREBRAIN_DEVELOPMENT
GO_MRNA_BINDING
GO_POSTSYNAPTIC_MEMBRANE
GO_REGIONALIZATION
GO_SYNAPTIC_MEMBRANE
Running Enrichment Score
Ranked list metric
Rank in Ordered Dataset

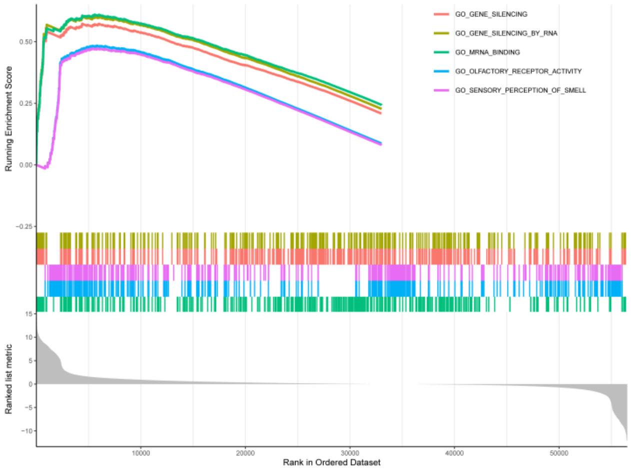
GO_GENE_SILENCING
GO_GENE_SILENCING_BY_RNA
GO_MRNA_BINDING
GO_OLFACTORY_RECEPTOR_ACTIVITY
GO_SENSORY_PERCEPTION_OF_SMELL
Running Enrichment Score
Ranked list metric
Rank in Ordered Dataset

Supplementary Figure 3. **DSS and DFI survival analysis and Cox proportional hazard regression analysis of target genes in Pan-cancer.**

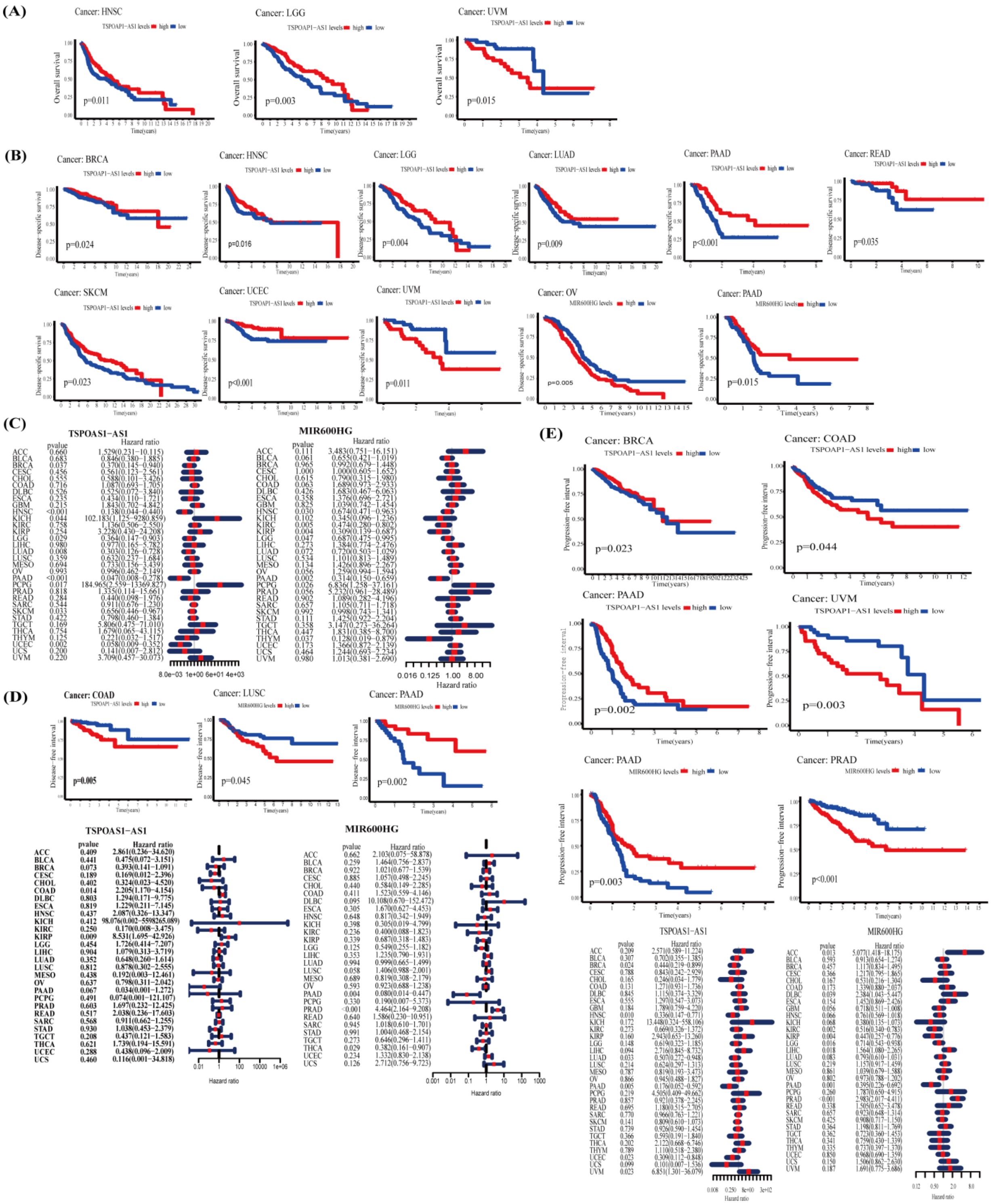

Supplementary Figure 4. Correlation between target genes and clinical stages of multiple cancers.

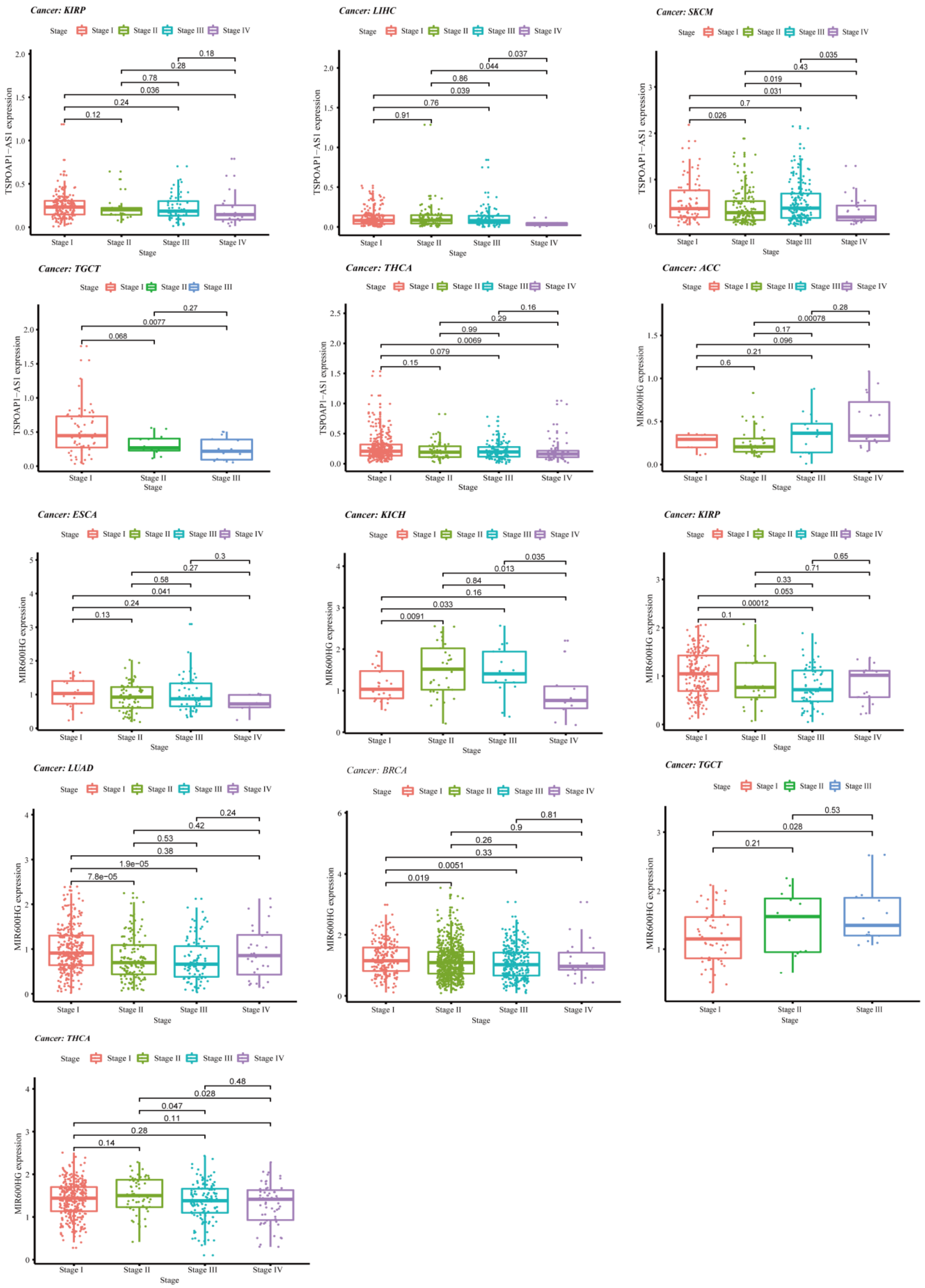

Supplementary Figure 5. Correlation of target genes with the content of stromal cells and immune cells in a variety of cancers.

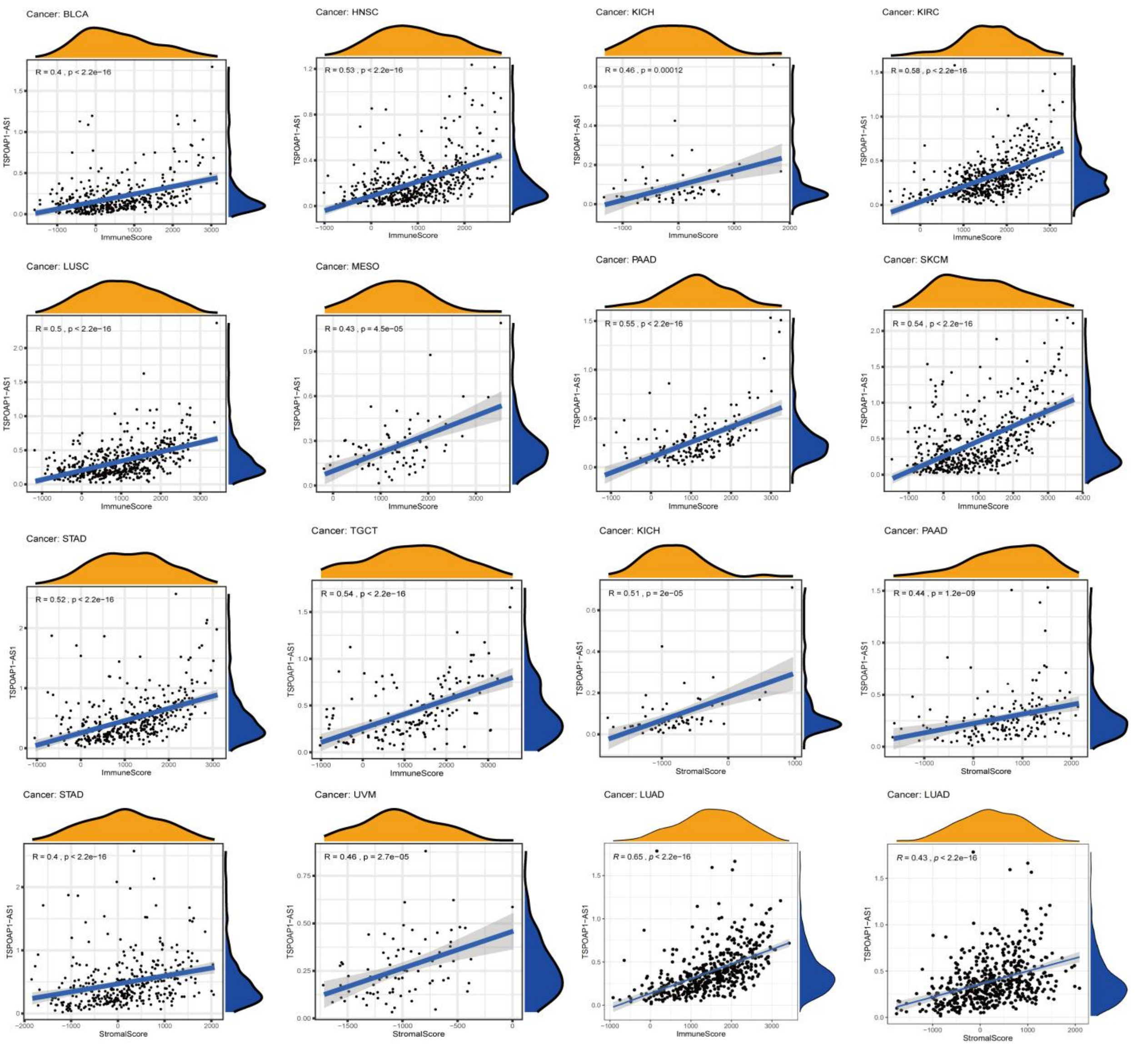

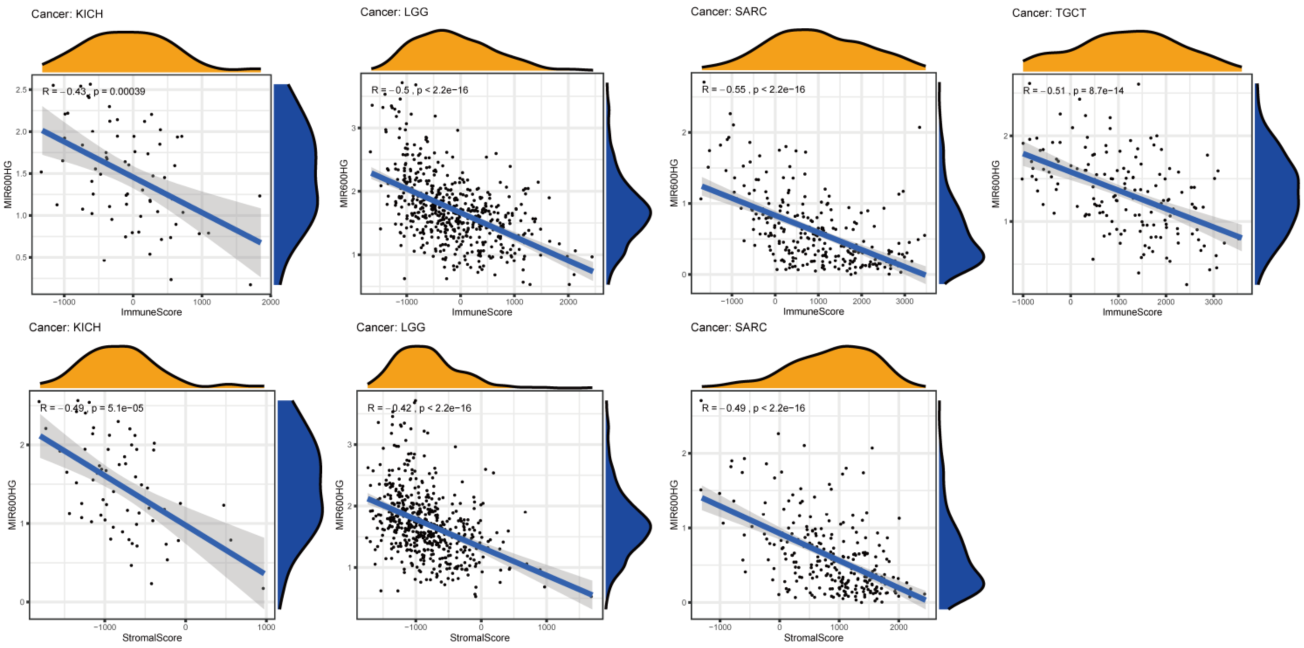

Cancer: KICH
R = −0.43 , p = 0.00039
MIR600HG
ImmuneScore
Cancer: LGG
R = −0.5 , p < 2.2e−16
MIR600HG
ImmuneScore
Cancer: SARC
R = −0.55 , p < 2.2e−16
MIR600HG
ImmuneScore
Cancer: TGCT
R = −0.51 , p = 8.7e−14
MIR600HG
ImmuneScore
Cancer: KICH
R = −0.49 , p = 5.1e−05
MIR600HG
StromalScore
Cancer: LGG
R = −0.42 , p < 2.2e−16
MIR600HG
StromalScore
Cancer: SARC
R = −0.49 , p < 2.2e−16
MIR600HG
StromalScore

Supplementary Figure 6. Correlation between target gene expression and immune cell infiltration in a variety of cancers.

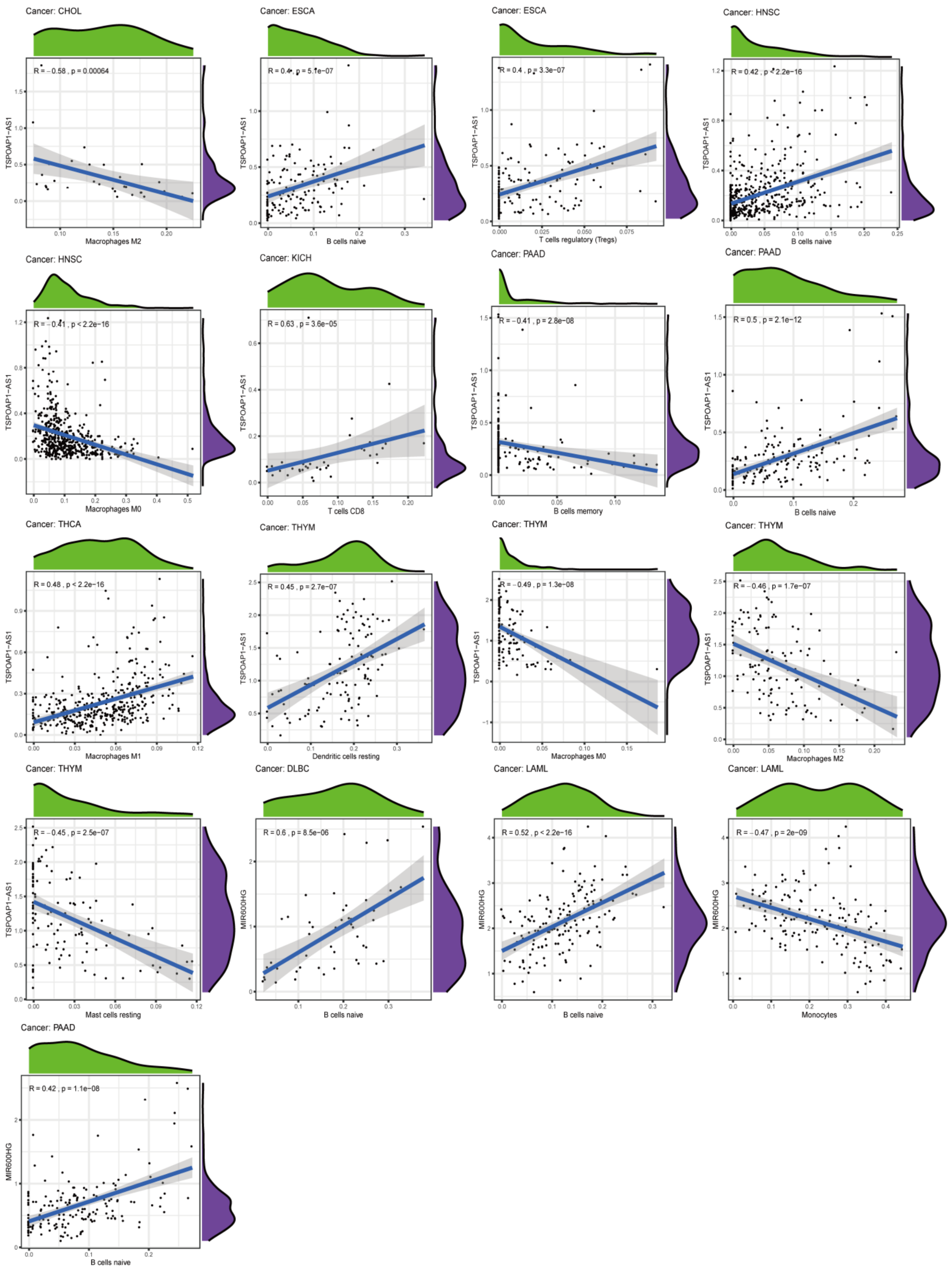

Supplement: Supplementary file 1 [file Data_Sheet_1.PDF]
